# Morpho-electrochemical behavior of particulate Zn electrodes revealed by X-ray and neutron tomography

**DOI:** 10.1038/s41598-026-49097-8

**Published:** 2026-09-02

**Authors:** Benedetto Bozzini, Francesco Casamichiela, Elisa Emanuele, Andrea Favalli, Riccardo Ferretti, Marc Looman, Andrea Pola, Nicola Sodini, Jacopo Strada, Stefano Vaccaro

**Affiliations:** 1 Department of Energy, Politecnico di Milano, via Lambruschini 4, 20156 Milano, Italy; 2 Joint Research Centre (JRC), European Commission, Via. E. Fermi, 21027 Ispra, Italy; 3 Elettra-Sincrotrone Trieste S.C.p.A., Basovizza, 34149 Trieste, Italy; 4 Department of Chemistry, Materials and Chemistry Engineering, Politecnico di Milano, via Mancinelli 7, 20131 Milano, Italy

**Keywords:** Chemistry, Energy science and technology, Materials science, Physics

## Abstract

Aqueous rechargeable Zn-based batteries are gaining attention for their sustainability, safety, and cost-effectiveness; however, no industrially viable solutions are yet available, due to the synergistic issues of passivation and shape change. In particular, flow batteries with particulate Zn anodes are attractive for long-term stationary storage, at the cost of additional morpho-chemical processes involving the Zn particle bed and the interparticle space (IPS). Thus, the understanding of this system requires space-dependent information about the arrangement, dimensions, and chemical states of Zn and its discharge products. To this aim, we employ dual-probe chemically sensitive X-ray and thermal neutron tomography and radiography. Specifically, we analyzed an in situ anode in both pristine and discharged states, and we followed its self-discharge process in operando. In situ X-ray tomography reveals the advancement of Zn particle consumption and IPS development. In situ neutron tomography shows the formation and distribution of hydroxides. In operando, neutron radiography gives access to hydrogen bubble formation and dynamics. This first-of-its-kind analysis provides crucial insights, directly informing anode design strategies. Our findings contribute to paving the way for next-generation Zn flow batteries with improved efficiency and longevity.

## Introduction

The challenge of electrochemical energy storage, ultimately implying independently powering electric vehicles, aircrafts, and robots, calls for batteries with reliability, durability, sustainability, and energy densities that are notably higher than those achievable with state-of-the-art Li-ion batteries (LIBs). To this aim, various post-Li technologies are being developed in parallel, among which batteries with Zn anodes, coupled with diverse, flexible cathode chemistries, represent a valid low-cost, non-toxic alternative^1,2^. Operationally flexible Zn-air fuel cells (ZAFCs), with flowing particulate Zn electrodes (PZE) coupled with oxygen cathodes, have been drawing attention for stationary storage applications due to their high theoretical energy density, low cost, ready and geopolitically diffuse source ability of the cheap and non-critical raw materials^3^ and safety. In principle, the intrinsic safety of ZAFCs is, in fact, not only due to the use of aqueous electrolytes but also to the possibility of de-powering the device using hydraulic and mechanical actions in addition to conventional electrical ones. A close-knit group of research groups^4–10^ and one developer^11^ are working on this technology, that, however, does not seem to have reached the commercial level. The promise of Zn anodes is nevertheless faced with many scientific and technological challenges related to the widely investigated, yet still poorly understood, dynamic morpho-chemistry of the Zn/electrolyte system. Due to the misleadingly simple stoichiometry of this system, combined with the relative ease of material preparation, phenomenological electrochemical testing, and traditional post-mortem microscopic and structural analyses, the controlling physicochemical complexities of electrode performance are difficult to identify and conceptualize. The key challenges of Zn electrodes in rechargeable batteries are passivation during discharge, the generation of unstable growth morphologies during charge, and parasitic hydrogen evolution during both discharge and charge. A recent overview of these issues can be found in the Supporting Information of^12^. The PZE, on the one hand, mitigates these issues because shape changes and chemistry can be reset to some extent by fluidizing the bed and transferring the partially corroded particles to a dedicated recharge reactor. On the other hand, during packed-bed operation, they are exacerbated by localization of reaction rate and reaction-product accumulation in the IPS, as well as by the formation of preferential electrolyte channels among particles. Thus, the availability of positive information regarding the morphology and composition of the IPS would be extremely valuable for a knowledge-based approach to Zn particulate anode optimization. Zn electrodes have been recently studied in battery-relevant conditions with various techniques (SEM, TEM, STXM, SPEM)^2,13^. Still, no definitive understanding of the aging and failure modes resulting from charge-discharge cycling has been achieved. Moreover, in the case of batteries with PZEs, the effects on performance of several parameters—including electrolyte flow rate, electrolyte concentration, and current density (c.d.)—have started to be studied^7,14–18^. However, systematic experimental testing and quantitative data treatment are still lacking, except for a close-knit group of electrochemical studies at the cell level that cannot rigorously discern the impact of the individual cell components^19–22^. In this scenario, imaging tools are a privileged route for providing information on the controlling factors of Zn anode performance. Specifically, in situ imaging can help pinpoint the physical bases of the impact of operating parameters that have so far been addressed exclusively from the point-of-view of the full-cell behavior: (i) temperature^6,23–25^, (ii) electrolyte composition and fluid dynamics^6,23–25^, (iii) design of anode chamber and current feeders^6^, (iv) air cathode structure^6,24,25^, and (v) cell aging^24^. In this scenario, the present work focuses on PZE for flow cells, aiming to assess the morphochemical evolution of the IPS. To this aim, we employ a dual-probe imaging approach that combines the strengths of X-ray and thermal neutron tomography^26^. An X-ray probe is ideal for characterizing the morphology of a battery component with high-Z materials. Thus, it enables a detailed description of Zn particle distribution, shape, and corrosion degree. At the same time, imaging with thermal neutrons (NI) offer element-selective sensitivity governed by nuclear interaction cross-sections, making it particularly effective for hydrogen-rich compounds even when embedded in metallic environments^27,28^. Relatively wide use of NI—primarily performed in “absorption contrast” mode with thermal or even cold neutrons—has been made for Li-ion battery studies, either as a separate tool or as a powerful technique complementary to X-ray imaging^29–32^. A wealth of studies has been produced from the static view of the 2D (radiography) and 3D (tomography) Li distribution^33,34^ to visualize the dynamic Li diffusion process and electrode expansion^35–37^, electrolyte consumption^38^, and gas evolution^39^. Regarding Zn anodes, so far, NI has been employed to study primary batteries^34,40^, but has never been used to track the spatial distribution of the H-containing Zn oxidation products and to dynamically monitor the aqueous electrolyte during battery operation or self-discharge processes. Thus, the morpho-chemical capabilities of combined X-ray and neutron tomography ensure a comprehensive in situ characterization of the state of a PZE, enabling advanced modeling tasks oriented toward device control in the framework of both the continuum equivalent-material approximation^41^ and the single-particle description^42^.

## Results

**Fig. 1:**
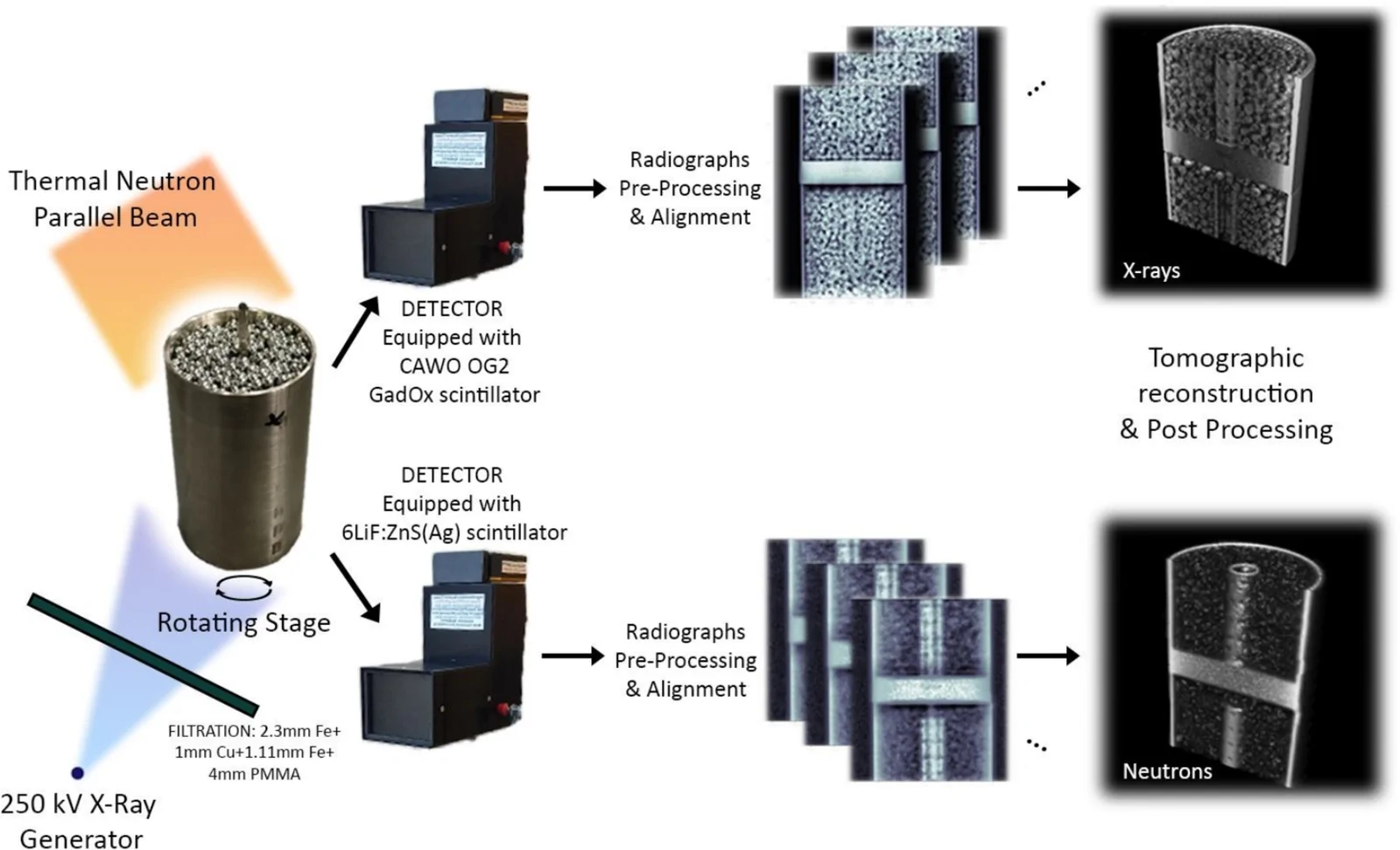
Flowchart of the dual-probe, thermal neutron and X-ray, tomography approach. From left to right, the sequence of operations is schematized as follows: irradiation with X-rays or neutrons, detection, radiography and data processing, and tomographic reconstruction. A collimated thermal neutron beam from the Triga research reactor was used for neutron irradiation. A cone beam configuration from an X-ray generator, equipped with an appropriate filter, was employed. A *400 μ m* 6LiF:ZnS(Ag) scintillator served as a neutron-to-light converter for thermal neutron imaging, while a CAWO OG2 GadOx scintillator was used for X-ray imaging. Radiographs were acquired at appropriate rotation angles and then combined for the tomographic reconstruction.

The flowchart in Fig. 1 outlines our experimental and analysis approach. We performed neutron and X-ray measurements of items consisting of two stacked cylindrical packed-bed PZEs for Zn flow cells, similar to the one shown in Fig. 1. The cell design was devised explicitly for imaging experiments, and simulates realistic operating conditions of a PZE. The body, fabricated with pure Ni, features an annular cylindrical compartment that houses the collapsing packed bed of Zn particles, hosting a KOH 6M aqueous electrolyte. A Ni rod is placed on the cylinder axis, serving as the counter-electrode to complete the electrochemical cell. Zn functions as the anodic active material, while Ni is chosen because it is the current-collector material of choice for an alkaline electrolyte and because it enables good neutron transmission at the energies considered and low activation. The cell dimensions, 30 mm in diameter, are typical of the particulate anode of a single Zn-air fuel cell stack cell. Zn spheroids of diameters in the 2–3 mm range are of practical interest and align with the imaging capabilities developed in this work. During the irradiation process, we stacked two cells with different depths of discharge (DOD): for details, see the “Methods” Section. This approach enables both to optimally utilize the available irradiation beam time and, importantly, directly compare two electrochemical states. Specifically, the bottom cell was in the pristine state, acting as a reference, while the upper cell underwent discharge, as detailed in the Subsection on Electrochemical Measurements of the Methods Section. In all experiments, identical Zn particulate cells were used for X-ray and neutron imaging: the pristine cell was imaged as-fabricated and later employed for the operando self-discharge test after electrolyte addition, while the discharged cell was drained and dried before both X-ray and neutron tomography measurements.

Cone-beam X-ray tomography measurements were performed at the European Commission’s Joint Research Centre (JRC) in Ispra, Italy, while thermal neutron measurements were carried out at the thermal neutron beamline at the TRIGA MARK II research reactor, located at the “Laboratorio di Energia Nucleare Applicata” (LENA) at the University of Pavia, Italy. Detection, data acquisition, pre-processing, calibration, and tomographic reconstruction were conducted using an in-house developed system (see “Methods” for details). Figure 1 presents a schematic representation of the experimental set-up, in which the sample is positioned on a rotating stage, and a series of lateral radiographs is obtained. The resulting tomographic reconstructed data comprise 3D voxel gray-scale images, with high- and low-attenuation materials appearing white and black, respectively. Post-processing, visualization, and analysis were performed using the Dragonfly software^43^.

### X-ray computed tomography

#### Morphology description

As an X-ray source, we utilized bremsstrahlung radiation produced by an X-ray tube set at 250 kV in voltage and in current with the set of X-ray filters removing the X-rays with energy less than about 60 keV from the spectrum (see Methods). The beam energy was set to 250 kV based on empirical observation of the exposure values of the radiographs, to utilize the full dynamic range offered by the sensor. The measurement consisted of acquiring a set of 360 lateral radiographs in one-degree steps, with an exposure time of 3 s per view, using a CAWO OG2 GadOx scintillator^44^. After data pre-processing and alignment, the tomographic reconstruction was performed using a 3D CUDA FBP implementation based on the ToMoBAR python package^45^.

**Fig. 2:**
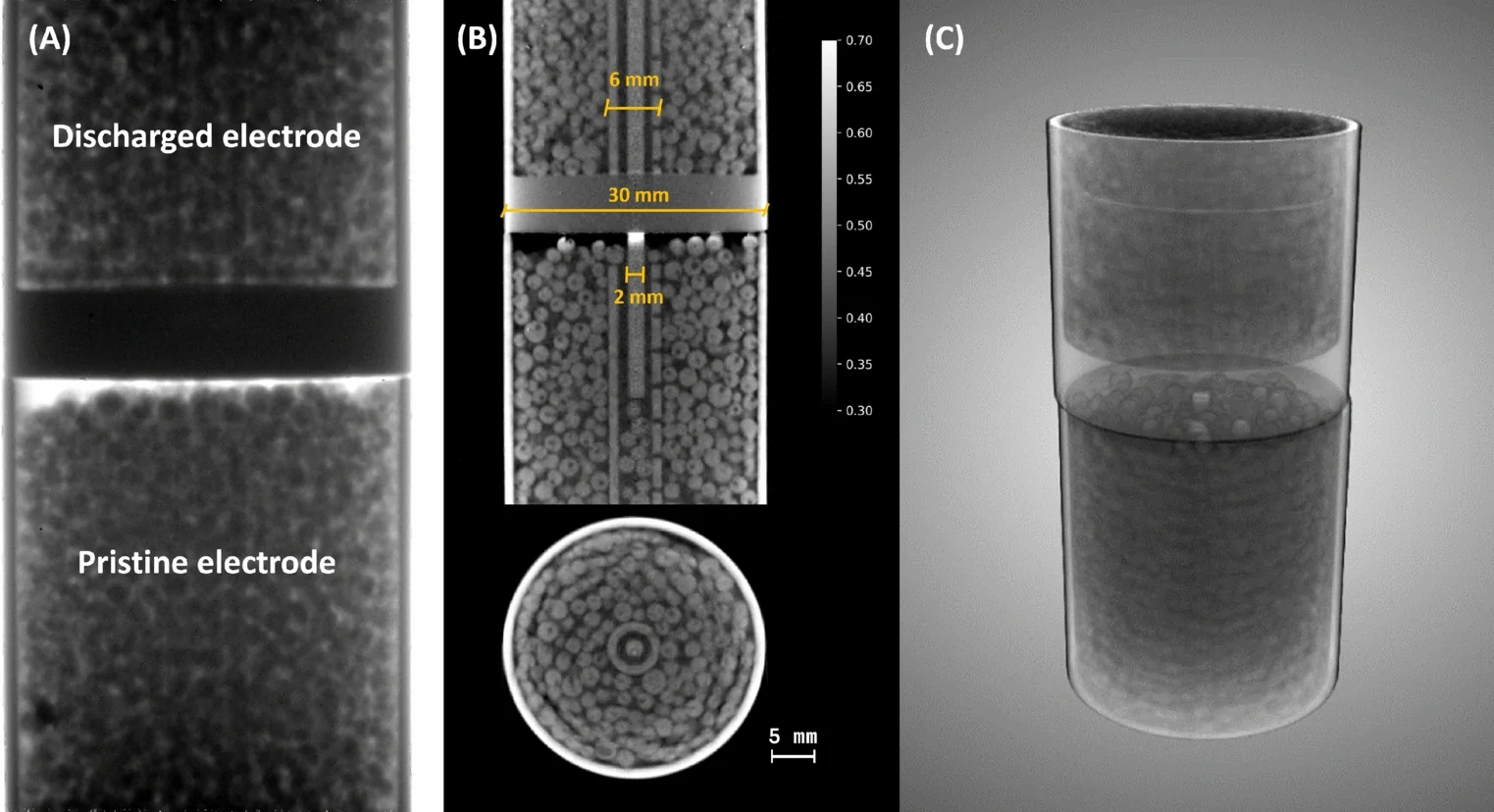
(**A**) Example of a single, post-processed X-ray lateral radiography; (**B**) Sagittal (top) and axial (bottom) slices of the 3D tomographic reconstruction. (**C**) 3D volume rendering of the tomographic reconstruction.

Figure 2 presents a single lateral radiograph and the results of the X-ray tomographic reconstruction: the bottom and upper cells are the pristine and discharge ones. X-ray tomographic imaging reveals the inner structure of the cells, providing details not accessible through standard X-ray radiography. Consistent with the estimated image resolution (see “Methods” section), features in the order of few hundred microns, such as spheroid cavities, can be distinguished. These features result from the rolling-based spheroid production process. When the hollow core is located in close proximity to the particle surface, the limited imaging resolution may prevent its separation from the IPS, resulting in segmentation artefacts with a characteristic concave profile in 2D cross-sections. Additionally, adjacent particles in close contact may occasionally be merged into a single segmented object, which is more pronounced in the discharged electrode, where Zn-containing corrosion products locally bridge adjacent particles, clogging the IPS.

#### Characterizing the inter-particle space (IPS)

**Fig. 3:**
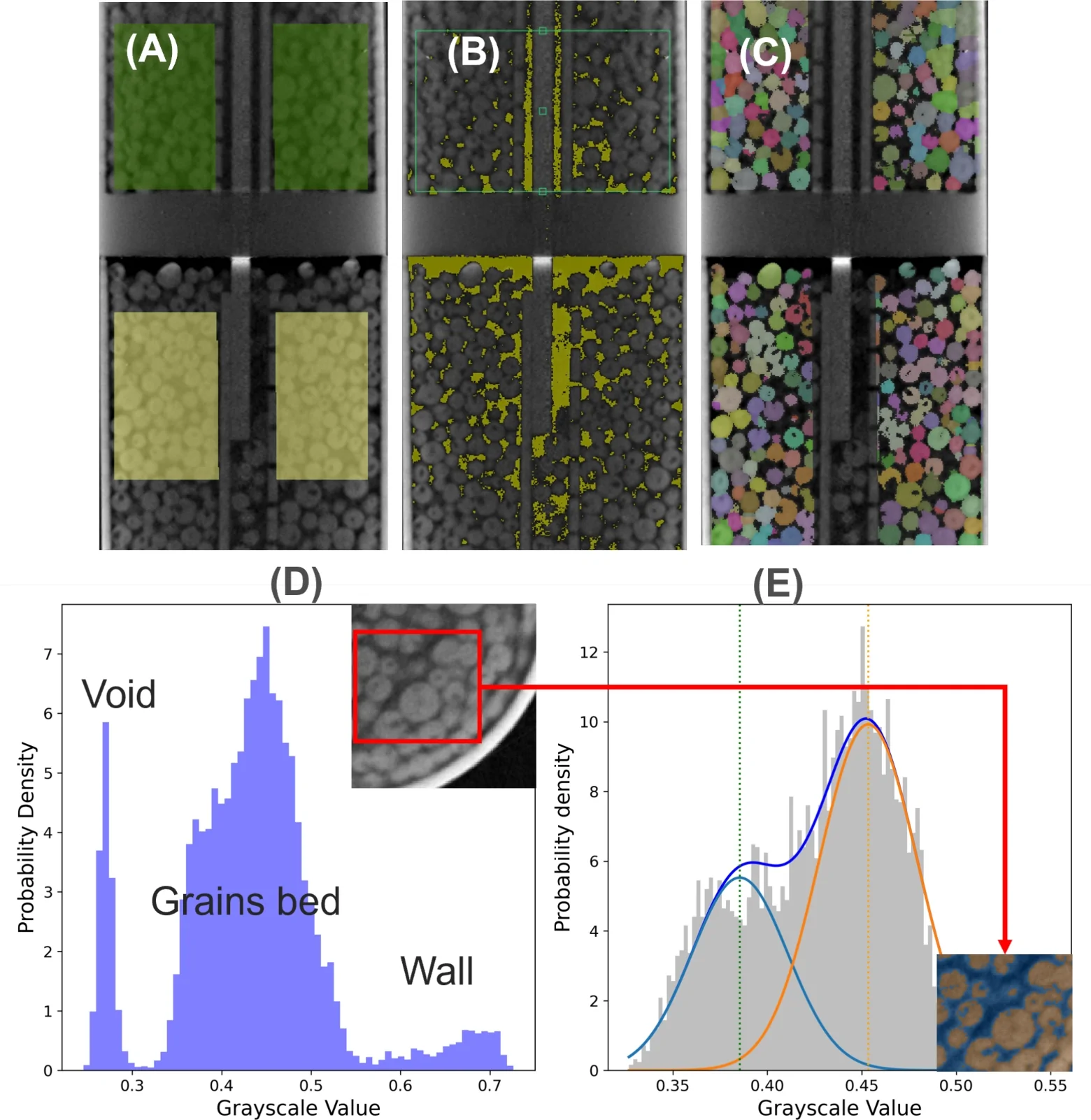
(**A**) Definition of cylindrical control volumes (highlighted with colored boxes) for the cycled (top) and pristine (bottom) cells. (**B**) Segmentation of the void inter-particle space using Otsu thresholding. (**C**) After segmenting the metallic grains, each individual particle is assigned to a distinct region of interest, enabling statistical analysis of morphological features across the particle population. (**D**) Histogram of pixel intensities for the image patch shown at the top right of the panel: the cell wall and background can be easily distinguished. (**E**) Pixel intensity distribution of the Zn particle bed region (central Ni rod excluded). The two-Gaussian fit (colored lines) resolves two components: interstitial void/IPS phase (orange, lower intensity) and metallic Zn grains (blue, higher intensity); dashed lines mark the Gaussian centroids used to define the segmentation threshold.

The porosity, which represents the free volume fraction that can accommodate the electrolyte, serves as a key parameter for quantifying changes in cell permeability to the electrolyte and release of discharge products from the spheroids to the electrolyte, from the pristine and the aged states. Figure 3A shows a sagittal slice of the stacked cells. First, a fixed-volume cylindrical control region (green and yellow regions) $V_{control}$ is defined for the pristine and discharged cells, encompassing only the Zn-spheroids bed and excluding the central rod and the external boundaries. Next, the void phase is segmented out by applying Otsu thresholding to the entire reconstructed volume (Fig. 3B); the cylindrical subvolumes defined in (A) are used solely as control volumes for porosity quantification, not as inputs to the thresholding algorithm. The void volume fraction is then calculated as $V_{void}/V_{control}$, where $V_{void}$ corresponds to the void phase volume in the aged or pristine cell. For an appropriate heuristic choice of imaging and segmentation parameters, the cycled and pristine cell exhibit a difference that can be attributed to corrosion processes. Of course, a full quantitative treatment is beyond the scope of the present work. Within this framework, we found void fractions respectively of ca. 6% and ca. 11% for the discharged and pristine cells, respectively. This finding is consistent with the smaller void volume developing in the discharged electrode, resulting from releasing oxidation products in the IPS.

#### Particle population morphology

The discharge process leads not only to the release of oxidation products into the IPS, but also to morphological variations of the Zn spheroids, that impact the electrochemical activity of the packed-bed electrode. Thus, it is highly relevant to investigate changes in the dimensions and shape of the spheroid population and in IPS connectivity. To this aim, we performed a pore analysis using the OpenPNM toolbox^46^, elaborating on grain segmentation. The user-defined segmentation threshold has been selected euristically on the basis of the voxel intensity distribution. While the background signal (i.e., the void phase) is clearly distinguishable and can be effectively segmented using the Otsu thresholding method (see Fig. 3D), the grains bed region pixel intensity distribution comprises the overlap of two components, corresponding respectively to the interstitial void phase (lower attenuation) and the metallic Zn grains (higher attenuation), as shown by the two-Gaussian fit in Fig. 3E. Based on a fit of this bivariate distribution using a two-gaussian interpolation function, where the normalized greyscale intensity is the independent variable, a threshold value of 0.43 was empirically determined to effectively separate the interstitials from the metal grains (see Fig. 3E). The thus segmented metallic phase was fed to OpenPNM, which assigns each spheroid to a distinct ROI (see Fig. 3C). This allowed the statistical analysis of shape and size for the spheroid population.

**Fig. 4:**
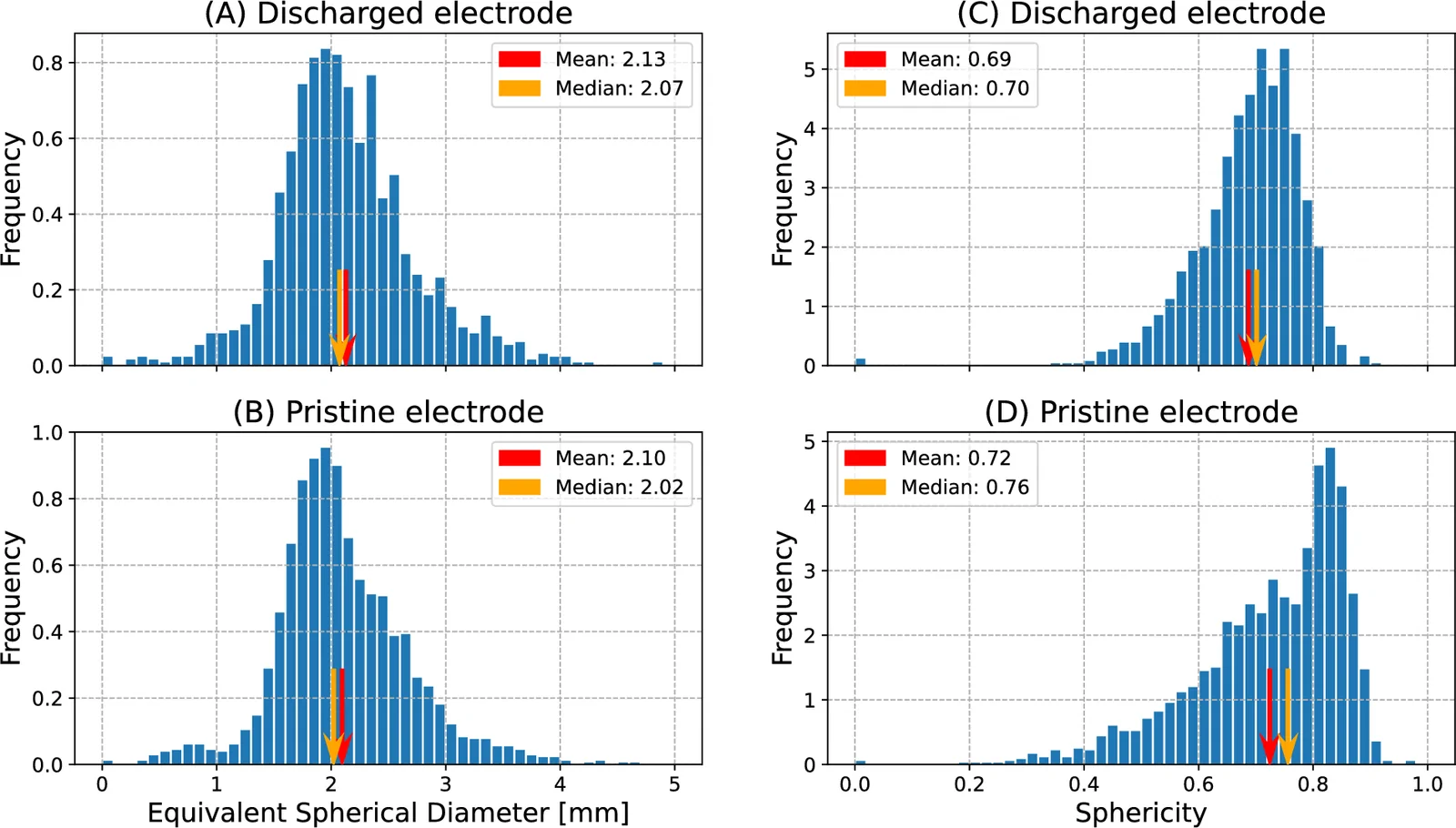
(**A**,**B**) Equivalent spherical diameter (see text). distribution over the grain population, with corresponding median, mean. (**C**,**D**) Sphericity distribution.

No measurable differences are observed in terms of equivalent diameter, which refers to the diameter of a hypothetical sphere with the same volume as the grain, between the two states. This is consistent with the Depth of Discharge (DOD) selected for this study (Fig. 4A,B). To gain a deeper insight into possible discharge-induced particle shape variations, we analyzed sphericity, defined as the ratio of the surface area of a sphere to the surface area of the particle: a value closer to 1 indicates that the particle closely resembles a sphere^47^. We observed that the discharged electrode exhibits a lower fraction of particles with high sphericity than the pristine one, coherently with the agglomeration of corroded particles, resulting from the formation of necks of the oxidation-product between the spheroids (Fig. 4C,D). In addition to particle size and shape estimation, we also evaluated the IPS connectivity. As far as IPS connectivity is concerned, we estimated it through the Euler Characteristic Number (ECN)^48^, defined from the counts of vertices (V), edges (E), and faces (F) of the segmented void phase as ECN = V - E + F. ECN > 0 corresponds to compact, simply connected objects; ECN = 0 to disconnected objects; ECN < 0 to objects containing loops and interconnected channels^49^. The highly negative ECN values reported here (− 8176 for pristine, − 5590 for discharged) indicate strongly interconnected void networks whose reduced magnitude upon discharge reflects IPS clogging by oxidation products; while absolute values depend on imaging resolution and segmentation parameters, their relative change provides a robust connectivity indicator. The ECN was evaluated using the same void segmentation and control volume detailed above. The higher IPS connectivity found in the pristine electrode confirms that oxidation product release leads to pore clogging.

### Neutron computed tomography

#### Radial distribution of H-containing Zn oxidation products

The PGNAA beamline at the LENA research reactor offers a collimated thermal neutron flux in the order of ${10^{7}}{\textrm{cm}^{-2}\textrm{s}^{-1}}$ at the shutter position, with a 5 cm diameter^50^.

**Fig. 5:**
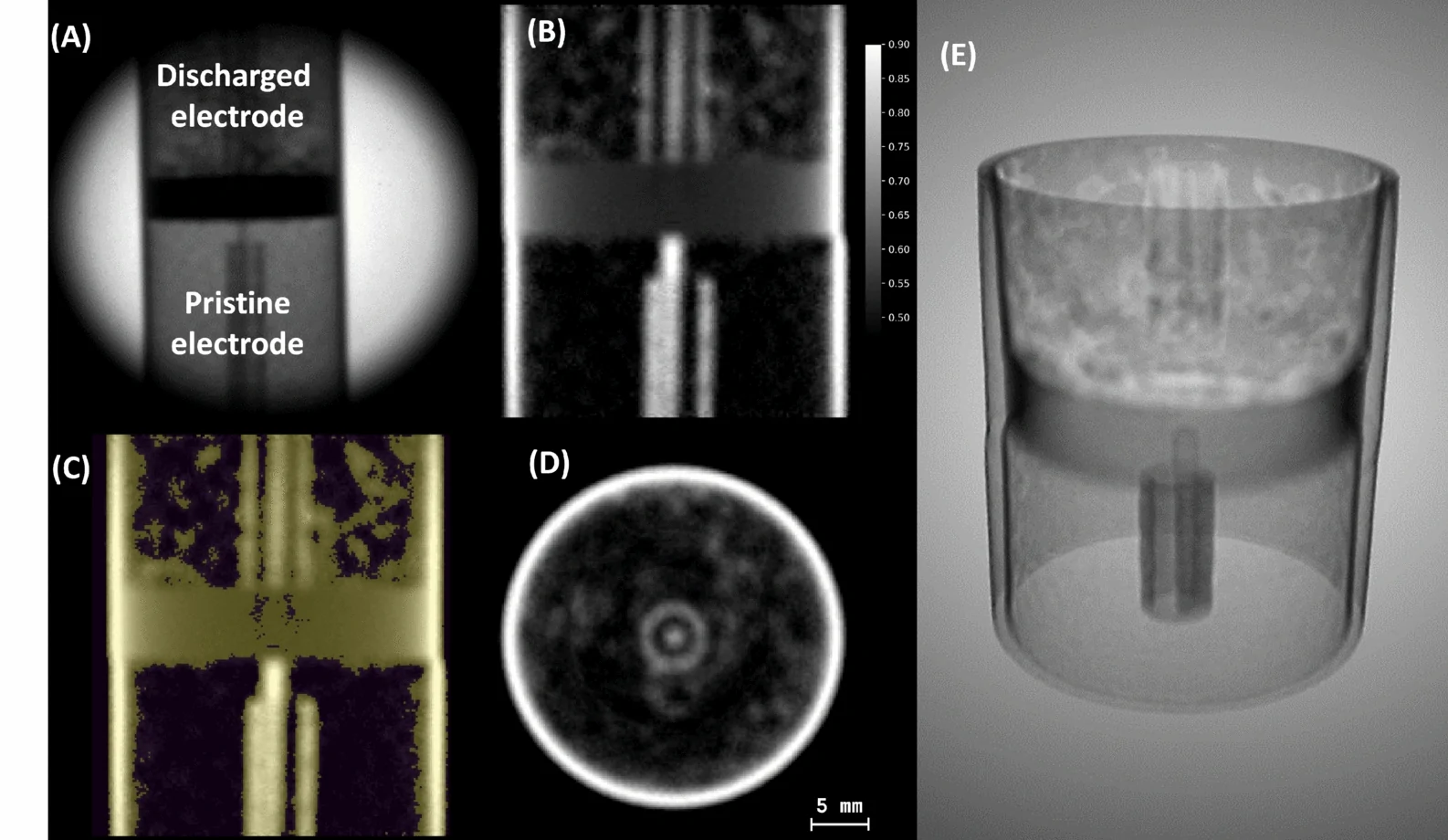
(**A**) Representative neutron radiography of the pristine and discharged electrodes. (**B**–**D**) Sagittal and axial slices of the 3D tomographic reconstruction. The light grey regions correspond to hydrogenated discharge products. The red segmentation in (**C**) indicates the most attenuating materials. (**E**) Volume rendering, highlighting hydrogenated species.

The tomographic measurement consisted of 120 lateral radiographs, acquired at 3-degree intervals, with an exposure time of 20 s per radiograph. Tomographic reconstruction was performed using a 3D CUDA FBP implementation based on ToMoBAR python package^45^. Figure 5A shows a lateral neutron radiograph: the opacity difference between the two electrodes is clearly visible. The discharged cell exhibits higher radiographic contrast, owing to the effect of hydroxides/hydrated oxides, generated electrochemically. This difference is even more evident in the tomographic reconstruction (Panels (B)–(E) in Fig. 5). In order to analyze their spatial distribution, we selected a segmentation threshold that would not yield feature separation in the pristine electrode. Specifically, normalizing in the 0–1 range the 3D image data, a value of 0.52 was heuristically selected. Given the neutron tomography spatial resolution ( 1.6–2 mm), individual Zn particles are not resolved; the high-attenuation segmented phase represents solid hydrogenated Zn oxidation products (hydroxides/hydrated oxides) within the IPS, as neutron measurements were performed after electrolyte drainage, rinsing, and drying to eliminate liquid KOH/water attenuation.

**Fig. 6:**
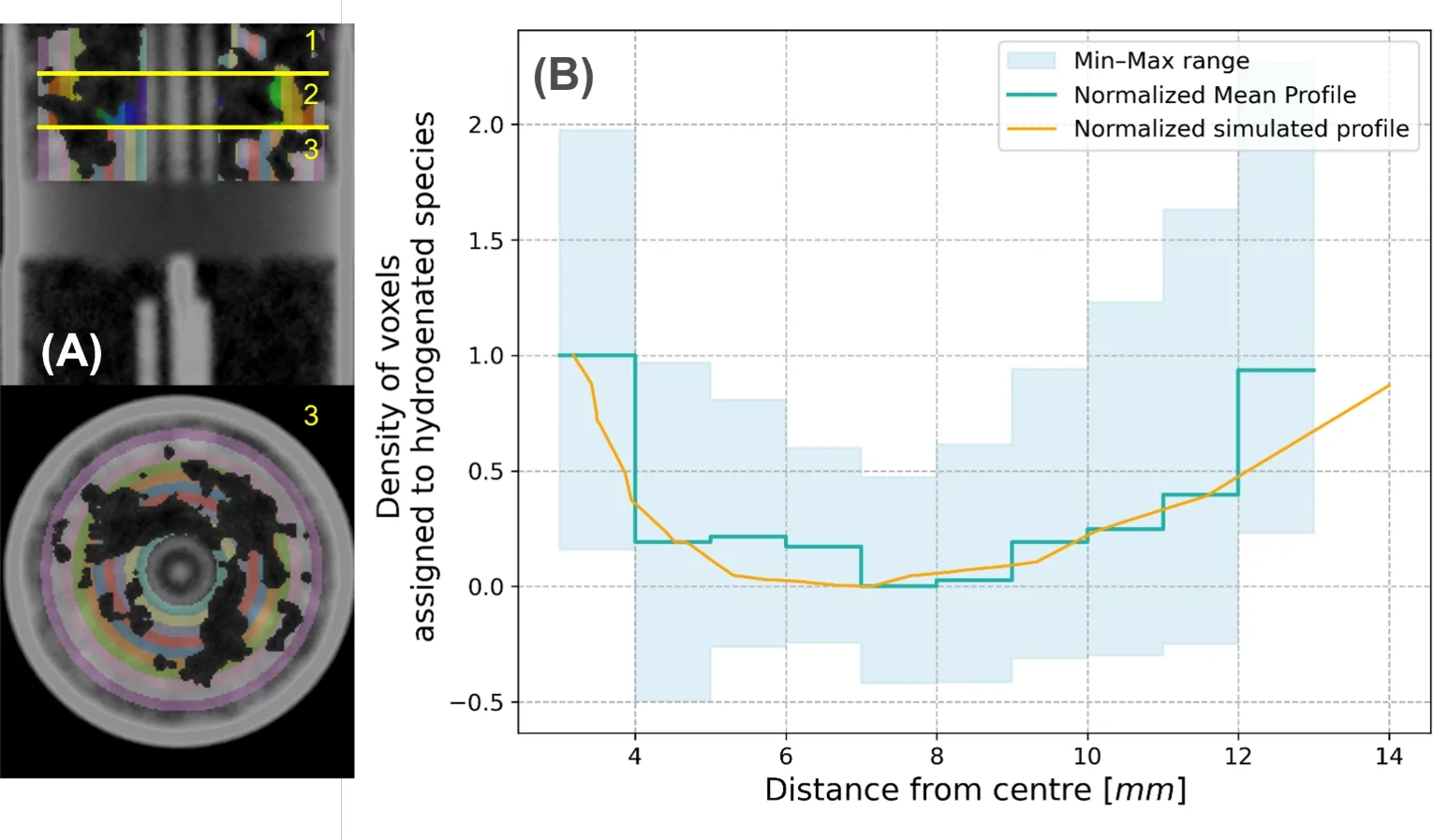
Radial distribution of hydrogenated discharge products. (**A**) Discretization of discharged electrode showing 3 delineated axial height regions (1 = top, 2 = middle, 3 = bottom) used for radial analysis, overlaid on sagittal neutron tomography slice. Darker regions represent low-attenuation phase (voids and metallic Zn); lighter regions indicate higher-attenuation materials in IPS. 10 radial shells are analyzed within each height region. The axial slice shows detail of region 3. (**B**) Experimental and simulated radial distribution of hydrogenated species (see text for details).

To estimate the radial distribution of hydrogenated species, we performed a discretization of the electrode volume, compatible with the available space resolution. Heuristically, we found that 10 cylindrical shells and 3 axial zones are appropriate to evaluate the absorption variability. A representative image of the discretized electrode is depicted in Fig. 6A. The volumetric segmented voxel density was computed at each radial distance and averaged over the three height regions: the variability was estimated as the minimum–maximum range: the results are plotted in Fig. 6B. The profile represents, for one electrode, the radial volumetric density of segmented voxels averaged over the three axial height regions, with the shaded area indicating the corresponding minimum–maximum range. It can be noticed that a minimum develops in the radial distribution. This feature can be straightforwardly rationalized in terms of electrochemical reaction-diffusion phenomena. Briefly, the precipitation of Zn(II) hydroxides/hydrated oxides is controlled by the evolution of the current density distribution, resulting from changes in ionic conductivity and ${\hbox {Zn}^{2+}}$ diffusivity driven by oxidation-product precipitation and in the electronic conductivity resulting from passive film formation. These results in progressive passivation of the Zn particles in the high-current density region, which confines the electrochemical reactivity towards the outer wall, thus leading to a steady state profile in which the concentration of Zn(II) hydroxides/hydrated oxides decreases from the external wall towards the central cathode compartment where free-electrolyte is present. A mathematical model of this process is reported and commented in the Supplementary Information and the computed profile is overlapped to the experimental one in Fig. 6B.

#### Self-discharge of the Zn anode

**Fig. 7:**
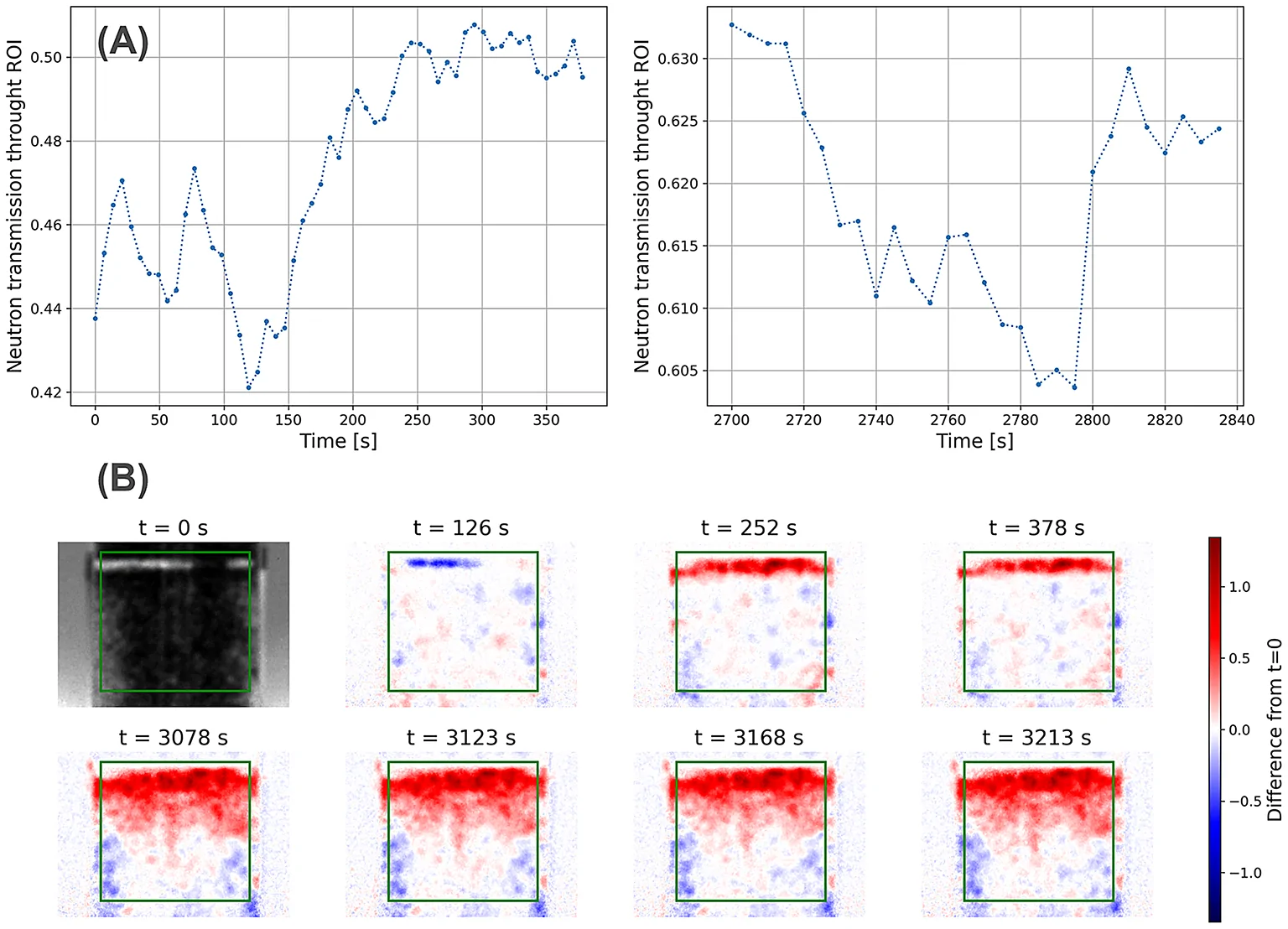
Electrode at open-circuit potential in contact with the electrolyte. (**A**) Time-resolved neutron transmission over the region of interest (ROI). The left panel shows the sequence immediately following the electrolyte injection, while the right panel corresponds to a sequence acquired after a 45-min resting period. The observed fluctuations are attributed to the formation and release of ${\hbox {H}_{2}}$ gas bubbles due to self-discharge. Over longer timescales, a saturation trend in transmission is observed, which reflects the gas accumulation near to the (close) interface. (**B**) Difference maps showing the variation in neutron transmission at different time points relative to the reference at t = 0. Red zones indicate regions of increased neutron transmission, highlighting a progressively stable and reduced electrolyte density near the interface, consistent with the trend observed in panel (**A**).

Neutron radiography enables the assessment of the distribution of hydrogen-containing compounds associated with both ${\hbox {Zn}^{2+}}$ release and ${\hbox {H}_{2}}$ gas formation during the self-discharge of the Zn anode. This process is of practical importance because, on the one hand, it leads to the loss of active metallic Zn through the formation of ${\hbox {Zn}^{2+}}$-containing species, and, on the other hand, it causes pressure to build up inside the battery due to ${\hbox {H}_{2}}$ accumulation. Once the radiographs are normalized with respect to the flat field, they can be considered as transmission maps. Figure 7 presents the study of the neutron transmission through the electrode at selected times after electrolyte introduction. Under open-circuit potential conditions, Zn is oxidized, and water is reduced, evolving ${\hbox {H}_{2}}$ bubbles Eq. (1). The spatial resolution of the radiographs allows for the observation of bubble formation and electrolyte evolution, while the average neutron transmission measured over a region of interest (ROI) reveals a progressive accumulation of gas in correspondence of the closed interface. Accordingly, over time the neutron transmission exhibits fluctuations due to abrupt changes in gas fraction caused by bubble evolution. On longer timescales, however, an overall increasing trend is observed (Fig. 7A). This behavior is further confirmed by the difference maps, obtained by subtracting the transmission at t = 0 from subsequent frames: the transmission stabilizes over time, indicating that the gas has been released and the electrolyte has settled at the bottom of the cell (Fig. 7B).

1 $$\begin{aligned} Zn + 2H_{2}O \rightarrow Zn(OH)_{2} + H_{2} \end{aligned}$$

2 $$\begin{aligned} Zn(OH)_2 + 2\,OH^- \rightarrow [Zn(OH)_4]^{2-} \end{aligned}$$

## Discussion

Our study demonstrates the potential of a dual-probe imaging approach, combining X-ray and thermal neutron tomography, for investigating the morphochemical evaluation of a particulate Zn bed anode. From an electrochemical viewpoint, we have demonstrated that tracking the joint evolution of the distribution of hydrogenated oxidation products and the morphological changes of both the Zn bed as a whole and the individual particles is possible. Moreover, ${\hbox {H}_{2}}$ evolution resulting from self-discharge can be monitored dynamically. Access to this type of information can be directly used to optimize both the design (hopper shape, particle arrangement, and degree of filling) and the management (consumption rate and distribution, electrolyte channeling, formation of hot spots) of particulate Zn bed electrodes (PZE). This finding has significant implications, as it informs the design of efficient and sustainable flow cells, aligning with the growing demand for reliable, low-cost, and environmentally friendly storage solutions. By providing unprecedented insights into the morphochemical evolution, our results pave the way for the design of the next generation flow cells with PZE. Our ongoing research is building upon our findings, along the following directions: (i) monitoring of electrolyte channeling patterns developing in experiments with flowing electrolyte; (ii) improvement in hopper-anode cell geometry, based on multiphysics modeling and (iii) assessment of the impact of electrolyte flow on hydroxide buildup in the IPS. Future studies will target the following aspects: (i) study of the recharge process, both in the same hopper-anode used for discharge or in a dedicated recharge reactor; (ii) monitoring and optimization of anode management policies involving the extraction of partially corroded and pristine particle feeding and (iii) systematic use of additives to control the buildup of hydroxides in the IPS.

## Methods

### Imaging system

**Fig. 8:**
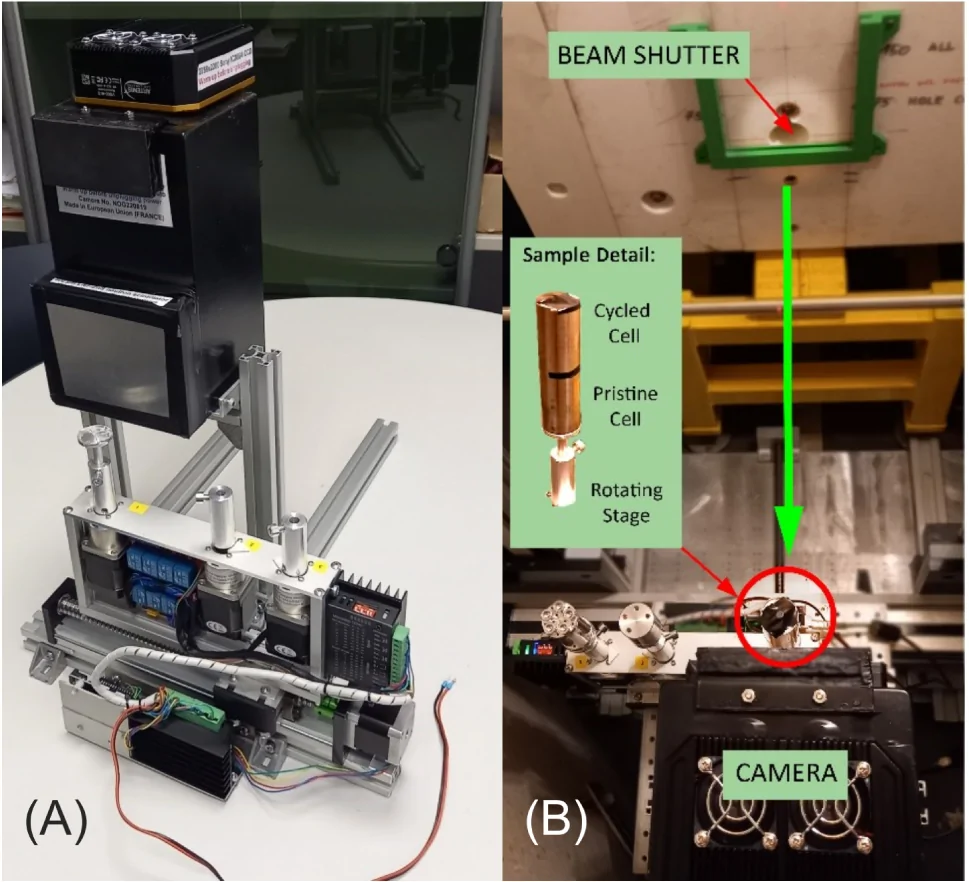
(**A**) Measurement system detail: imaging camera and sample positioning stage. (**B**) Picture of the irradiation set-up at the LENA neutron source.

#### Hardware

The imaging camera system, manufactured by Neutron Optics Inc., converts the impinging radiation field into an optical image using a scintillator screen. We used a CAWO OG2 GADOX screen for X-rays and a ${400}\;{{\upmu }{m}} {^{6}\hbox {LiF}}/\hbox {ZnS(Ag)}$ screen for thermal neutrons. The optical image is de-magnified by a photographic lens and focused on the surface of a CCD sensor for acquisition. The CCD sensor is a 2759 *×* 2200 SONY ICX694 CCD coupled with an electronic cooling system to reduce thermal noise. To prevent radiation damage, the CCD sensor is mounted at a 90-degree angle to the beam direction, while a 45-degree mirror directs the photon beam from the converter screen to the CCD sensor. Lead shielding is positioned to minimize the potential direct interaction of radiation with the sensor. The imaging system is connected to a sample positioning system, enabling the management of measurements for up to three samples without requiring access to the irradiation system. Specifically, a motorized translator stage is coupled with three rotating stages. A void position is left for flat-field acquisition Fig. 8.

#### Acquisition and pre-processing

The selected image binning is *2× 2* to reduce exposure time, data dimensionality and the computational time required for pre-processing and tomographic reconstruction. The pixel sizes, derived from features of known dimensions, are 108 *μ*m and 180 *μ*m for X-ray and neutron imaging, respectively. A set of 10 flat frames is acquired and median-stacked to produce a “master flat frame”, which corrects for field inhomogeneities. A comprehensive overview of the noise rejection and image calibration techniques used is provided in^51^, including bias correction, dark subtraction, flat calibration, and sigma clipping. Additionally, direct energy deposition by neutrons and X-rays within the sensor results in visible radiation tracks in the acquired images caused by ionization processes. To filter out radiation-fired pixels, we adopted the approach used for cosmic ray rejection in astrophotography, detailed in^52^. After pre-processing, rotation axis tilt calibration is performed using the algorithm described in^53^. As a final pre-processing step, each radiograph is treated as a numerical matrix of pixel values, and the negative logarithm of each entry is computed. This process linearizes the data to the effective attenuation coefficient, following the Lambert-Beer attenuation law.

#### Spatial Resolution of the neutron and X-ray radiographic system

We extracted the spatial resolutions for the X-ray and neutron radiography, as the full width at half maximum (FWHM) of the Line Spread Function (LSF) and from the Modulation Transfer Function (MTF). FWHM and MTF are calculated starting from the straight edge image, the average profile of which is the Edge Spread Function. We followed the approach described in^54^, adopting a 10% threshold for the definition of the limiting spatial frequency $f_{limit}\,[cycles/mm]$^55^. According to this approach, the spatial resolutions of the X-ray and neutron radiography systems were estimated to be approximately in the range 0.5–0.6 mm and 1.6–2 mm, respectively. These should be considered as lower bounds for the tomography resolution.

### Cell design

**Fig. 9:**
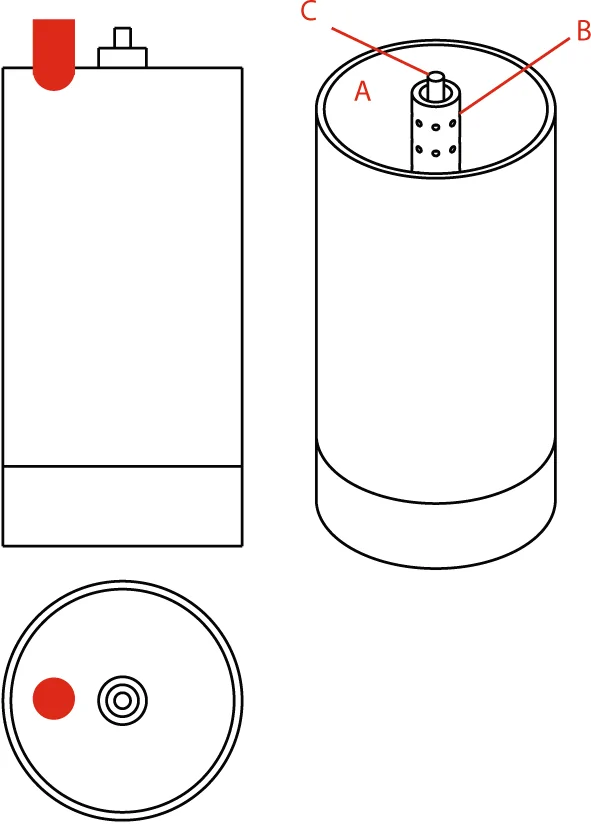
Cell design details. (**A**) Annular cylindrical compartment hosting the Zn particle bed; (**B**) perforated 6 mm Ni cylinder separating the Zn particle bed from the free-electrolyte compartment; (**C**) 2 mm Ni rod acting as counter-electrode. The reference electrode position is marked in red. The outer cylinder is 50 mm in height and 30 mm in outer diameter.

Figure 9 shows the dimensions in mm of all the sub-components of the cell, while also providing the position of the reference electrode, which is considered for the numerical simulation (details in the Supplementary Information).

### Electrochemical measurements

**Fig. 10:**
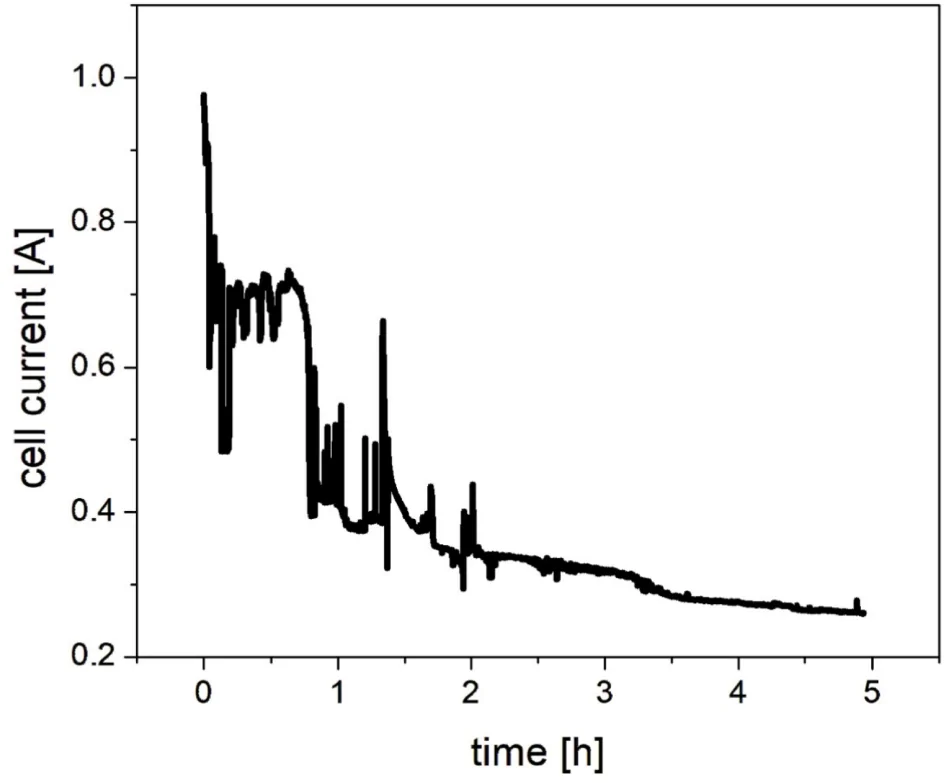
Cell current evolution of the ZPE polarized potentiostatically at 50 mV vs time.

Electrochemical measurements, consisting in anode discharge experiments, were carried out with a VersaSTAT 3 potentiostat in potentiostatic mode with a three-electrode configuration, using an Hg/HgO reference electrode collocated in the position depicted in red in Fig. 9, i.e. in contact with the electrolyte at the top of the Zn particle bed in halfway between the external cell wall and the perforated cylinder B. A three-electrode configuration was adopted because the Zn anode discharge experiments were carried out in a half-cell setup, using graphite as the counter electrode and operating under potentiostatic control. Under these conditions, the presence of a reference electrode was necessary to accurately monitor and control the potential of the Zn anode independently of the counter electrode contribution. Regarding its positioning, the reference electrode was placed as close as possible to the bed of Zn particles in order to minimize the uncompensated resistance and therefore reduce the ohmic drop (iR drop) between the working electrode and the reference electrode. This configuration ensured a more reliable measurement of the actual anode potential during discharge. We applied an anodic polarization of 50 mV anodic with respect to the measured ${\textrm{Zn}/{\textrm{Zn})_{2}}^{+}}$ open-circuit potential for 5 hours, corresponding to a DOD of ca. 17%. The reason for this choice is to inject a sizable amount of oxidation product injection into the IPS without significantly affecting the Zn particle dimensions. Moreover, at DOD of this level is practically representative for bulk Zn anodes, even though it can be pushed to higher values with PZEs. The resulting current profile is reported in Fig. 10. The current exhibits a characteristic progressive decrease, due to the accumulation of reaction products in the packed bed of Zn particles, in particular precipitation of zincates, that leads to partial clogging of the interparticle electrolyte and passivation. In practical working conditions, better activity of the anode can be achieved by circulating the electrolyte across the ZRE^7,10^. At the end of the discharge, in order to remove highly neutron-absorbing water and potassium, the cell was drained of the electrolyte, rinsed with distilled water until the rinse water was found to be neutral and dried by heating at $60^{\circ }$C in a muffle furnace overnight.

### Irradiation facilities

#### Thermal neutrons

Thermal neutron measurements were performed at the prompt-gamma thermal neutron beamline (PGNAA) at the TRIGA MARK II research reactor, located at the “Laboratorio di Energia Nucleare Applicata” (LENA) at the University of Pavia, Italy. The PGNAA line provides a collimated thermal neutron beam with a diameter of 5 cm.

#### X-ray irradiation facility

We conducted measurements at the X-ray Digital Radiography System (XDRS) facility at the JRC Ispra in Italy. The X-ray system features a Comet MXR-451/26 bipolar (two power supplies) metal-ceramic, oil-cooled X-ray tube, equipped with a tungsten anode and a 30-degree takeoff angle. The system has high voltage supplies with opposite polarity, and operates at voltages ranging from 100 up to 450 kV (maximum power 4.5 kW). The inherent filter stack consists of materials in the following order: 2.3 mm Fe, 1 mm thick Cu, 1.11 mm Fe, and 4 mm PMMA. The X-ray bremsstrahlung spectrum for an operating high voltage of 250 kV was calculated using SpekPy^56^. The filtered spectrum has a lower cut-off energy of approximately 60 keV (calculated as 1% of the total signal in the cumulative X-ray spectrum) and extends to 250 keV (the endpoint). The average energy of the X-ray bremsstrahlung spectrum was estimated to be 135 keV.

## Supplementary Information

## Data Availability

The data that support the findings of this study are available upon reasonable request. Due to the large size of the imaging datasets and the need for specialized reconstruction and analysis workflows for correct interpretation, the full datasets are not currently deposited in a public repository.
