# Supplementary material for: Morpho-electrochemical behavior of particulate Zn electrodes revealed by X-ray and neutron tomography
Source: Sci Rep. 2026 Sep 2;16:27516. doi: 10.1038/s41598-026-49097-8 (PMC13538399; doi:10.1038/s41598-026-49097-8)
Supplement: Supplementary file 1 [file 41598_2026_49097_MOESM1_ESM.pdf]

Supplementary Information

The electrochemical zincate distribution model

1. *Domain description*

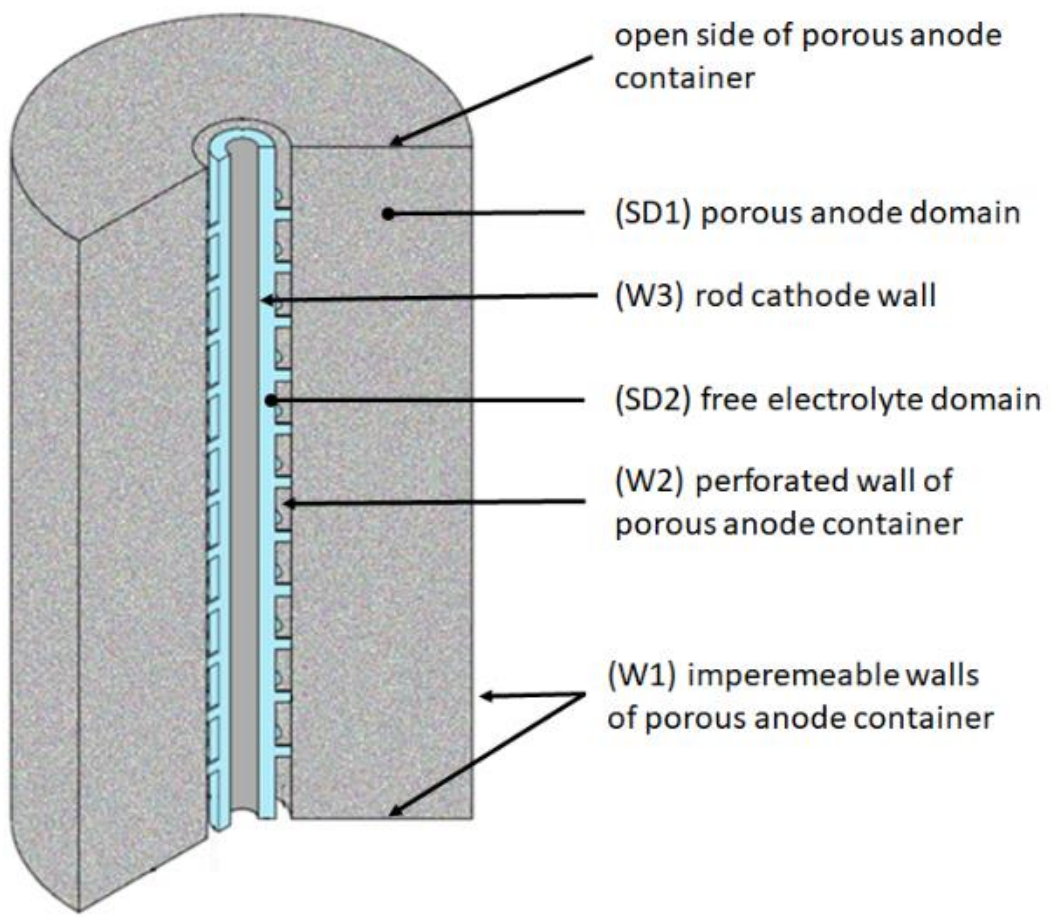

Figure 11: The integration domain for the electrochemical model representing the experimental cell.

The 3D domain modeling of the electrochemical cell employed for the experiments is depicted in Figure 11: The integration domain for the electrochemical model representing the experimental cell. and is devised as follows. (Subdomain SD1) The particulate anode is modeled with the porous electrode approach (see, e.g., [1]), consisting of one subdomain in which the charge conservation equations are solved for both the metal and the electrolyte phases, and the local electrochemical potential is defined as the difference of the local potential of the metal and electrolyte phases (see details in Section 3 below). In this sub-domain, the chemical species conservation equations are defined for zincates and hydroxide (see details in Section 2 below). The borders of the porous anode domain and the rest of the cell description match exactly the real cell geometry. In particular, the porous anode subdomain is confined by continuous, impermeable walls (W1) representing the external and lower Ni wall of the cell, by a perforated wall (W2), giving access to the free electrolyte subdomain (SD2), that is, in turn, confined by the surface of the rod cathode (W3). The dimensions of the individual domains are the same as in the experimental cell.

2. Species conservation in the electrolyte

$$\nabla\left(-D_{i,k}\nabla c_{i,k} - z_i u_{m,i,k} F c_{i,k} \nabla \varphi_k\right) = R_{i,k}$$

Eq. S1

where i = $Zn(OH)_4^{2-}$, OH^-, and k = el (electrolyte), PA (porous anode). $c_{i,k}$ and $D_{i,k}$ are the concentrations and diffusion coefficients of the species i in domain k. z_i and $u_{m,i,k}$ are the valency and the mobility of species i in domain k, respectively, and F is the Faraday constant. The mobility is obtained from the diffusion coefficients with the Nernst-Einstein relation:

$$u_{m,i,k} = \frac{D_{i,k}}{RT}$$

Eq. S2

For the electrolyte in the porous anode, in Eq.s S1, S2 we employ the effective diffusion coefficient, obtained by correcting for the porosity e with the Bruggemann relation:

$$D_{i,PA} = D_{i,el}\varepsilon^{3/2}$$

Eq. S3

$R_{i,k}$ in Eq. S1 is the electrochemical reaction rate of species i in domain k, which will be detailed in Section 5 below.

The BCs for species conservation are:

$$-\mathbf{n} \cdot \mathbf{j}_{\mathbf{i,k}} = 0 \quad \text{no flux}$$

$$c_{i,k} \text{ continuity in el} - \text{PA interface}$$

Eq.S4

The electrochemical reactions that take place at the rod surface are described by the BCs in the form of electrode reactions:

$$-\mathbf{n} \cdot \mathbf{j}_{Zn(OH)_4^{2-},el} = \frac{\nu_{Zn(OH)_4^{2-},2} i_{cat}}{nF}$$

$$-\mathbf{n} \cdot \mathbf{j}_{OH^-,el} = \frac{\nu_{OH^-,2} i_{cat}}{nF}$$

Eq. S5

The current densities i_{an} and i_{cat} are defined in Eq. S11.

3. Charge conservation

Charge conservation is expressed by:

$$\nabla \cdot \mathbf{i}_l + \nabla \cdot \mathbf{i}_s = 0$$

Eq. S6

where the subscripts *l*, *s* represent the electrolyte and electrode domains, respectively. The Ohm law describes the current density in the electrode and electrolyte.

$$\begin{cases} \mathbf{i}_l = -\sigma_l \nabla \Phi_l \\ \mathbf{i}_s = -\sigma_s \nabla \Phi_s \end{cases}$$

Eq. S7

The current density *i* imposed on the rod has been chosen so that, at the center of the top of the porous anode domain, the potential difference between the electrode and electrolyte phases is 50 mV, coherently with the experimental control conditions: numerical values are reported in Table 1.

To model the porous electrode, the charge-conservation equations are:

$$\nabla \cdot \mathbf{i}_l = A_v i_{an}$$

$$\nabla \cdot \mathbf{i}_s = -A_v i_{an}$$

Eq. S8

where A_v is the specific surface area, and i_{an} described by the Eq. S11.

The reaction rate at an interface between the rod and an electrolyte corresponds to the normal current flux:

$$\mathbf{i}_l \cdot \mathbf{n} = i_{cat}$$

Eq. 9

where i_{cat} is given by the Butler-Volmer equation, described by the Eq. S11.

The boundary conditions for the charge conservation problem are discussed in Section 5.

4. Electrochemical reactions

The electrochemical reactions at the electrodes are the corrosion/plating of zinc.

$$Zn(OH)_4^{2-} + 2e^- \leftrightarrow Zn + 4OH^-$$

Eq. S10

The corresponding electrokinetics is described by the Butler-Volmer equation corrected for reagent and products concentrations.

$$\mathrm{i} = A_v \mathrm{i}_{0,\mathrm{Zn}} \left[\frac{c_{\mathrm{Zn(OH)}_4^{2-}}}{c_{0,\mathrm{Zn(OH)}_4^{2-}}} \exp\left(\frac{a_{a,\mathrm{Zn}} F \eta_{\mathrm{Zn}}}{RT}\right) - \left(\frac{c_{\mathrm{OH}^-}}{c_{0,\mathrm{OH}^-}}\right)^4 \exp\left(\frac{-a_{c,\mathrm{Zn}} F \eta_{\mathrm{Zn}}}{RT}\right) \right]$$

Eq. S11

where, the initial values $c_{\mathrm{Zn(OH)}_4^{2-}}(x, y, z, t = 0)$ and c_{OH^-} (x,y,z,t = 0) are used at the reference values $c_{0,\mathrm{Zn(OH)}_4^{2-}}$ and c_{0,OH^-}. $i_{0,Zn}$ is the exchange current density, $a_{a,Zn}$ and $a_{c,Zn}$ transfer coefficients of zinc, A_v the specific surface area, and η_{Zn} is the overpotential, expressed as:

$$\eta_{\mathrm{Zn}} = \Phi_s - \Phi_l - E_{eq,\mathrm{Zn}}$$

Eq. S12

where the equilibrium value $E_{eq,Zn}$ is given by the Nernst equation:

$$E_{eq,\mathrm{Zn}} = E^0_{eq,\mathrm{Zn}} - \frac{RT}{nF} \ln \left[\frac{c_{\mathrm{Zn(OH)}_4^{2-}} / c_{0,\mathrm{Zn(OH)}_4^{2-}}}{\left(c_{\mathrm{OH}^-} / c_{0,\mathrm{OH}^-}\right)^4} \right]$$

Eq. S13

Anodic(ian) and cathodic (icat) current densities are obtained in correspondence of positive and negative values of the h_{Zn}, respectively.

5. Solution strategy and choice of electrochemical reaction terms and electrical boundary conditions

As witnessed by the experimental data of Figure 10, the long-term electrochemical response of the cell under the applied polarization conditions is controlled by the progressive passivation of the particulate anode resulting from zincate precipitation.

In the present study, we resorted to an approximate treatment of this process, able to capture the main qualitative chemical features disclosed by neutron tomography: the model will be refined in follow-up work. The approximation adopted is that of running the simulation in two steps. (i) We initially run the transient-state version of the model with constant potential applied to all the boundaries of the solid phase of the particulate anode domain – representing a fully active electrode -, until the zincate concentration reaches the salutation limit in the region of the particulate anode extending for 20% of the radius and then close to the border. This first set of boundary conditions – detailed below in

Section 5.1 – yield a zincate concentration profile with a maximum inside the particulate anode, close to the free-electrolyte domain. (ii) Then, we run the steady-state version of the model with anodic electrochemical reaction and anodic potential applied only to the external border of the electrode, representing partial passivation of the node caused by zincate precipitation. This second set of boundary conditions – detailed below in Section 5.2 – yield a sloping zincate concentration profile growing towards the outer border of the particulate electrode domain.

5.1 Electrochemical reaction terms and electrical boundary conditions for the transient-state model

$R_{i,k}$ in Eq. S1 is the electrochemical reaction rate of species *i* in domain *k*, defined, in the transient-state model as:

$$\begin{cases} R_{i,el} = 0 \\ R_{Zn(OH)_4^{2-},PA} = -\dfrac{\nu_{Zn(OH)_4^{2-},1} i_{an}}{nF} \\ R_{OH^-,PA} = -\dfrac{\nu_{OH^-,1} i_{an}}{nF} \end{cases}$$

Eq. S14

The BCs for the charge-conservation problem are set as:

$$\begin{cases} -\mathbf{n}\cdot\mathbf{i}_l = 0, \quad -\mathbf{n}\cdot\mathbf{i}_s = 0 \quad \text{insulation} \\ \Phi = \Phi_0 \quad \text{porous anode walls} \\ \mathbf{n}\cdot\mathbf{i}_s = i_0 \quad \text{rod surface} \end{cases}$$

Eq. S15

Electrochemical reaction terms and electrical boundary conditions for the transient-state model are depicted in Figure 12 and Figure 13, respectively.

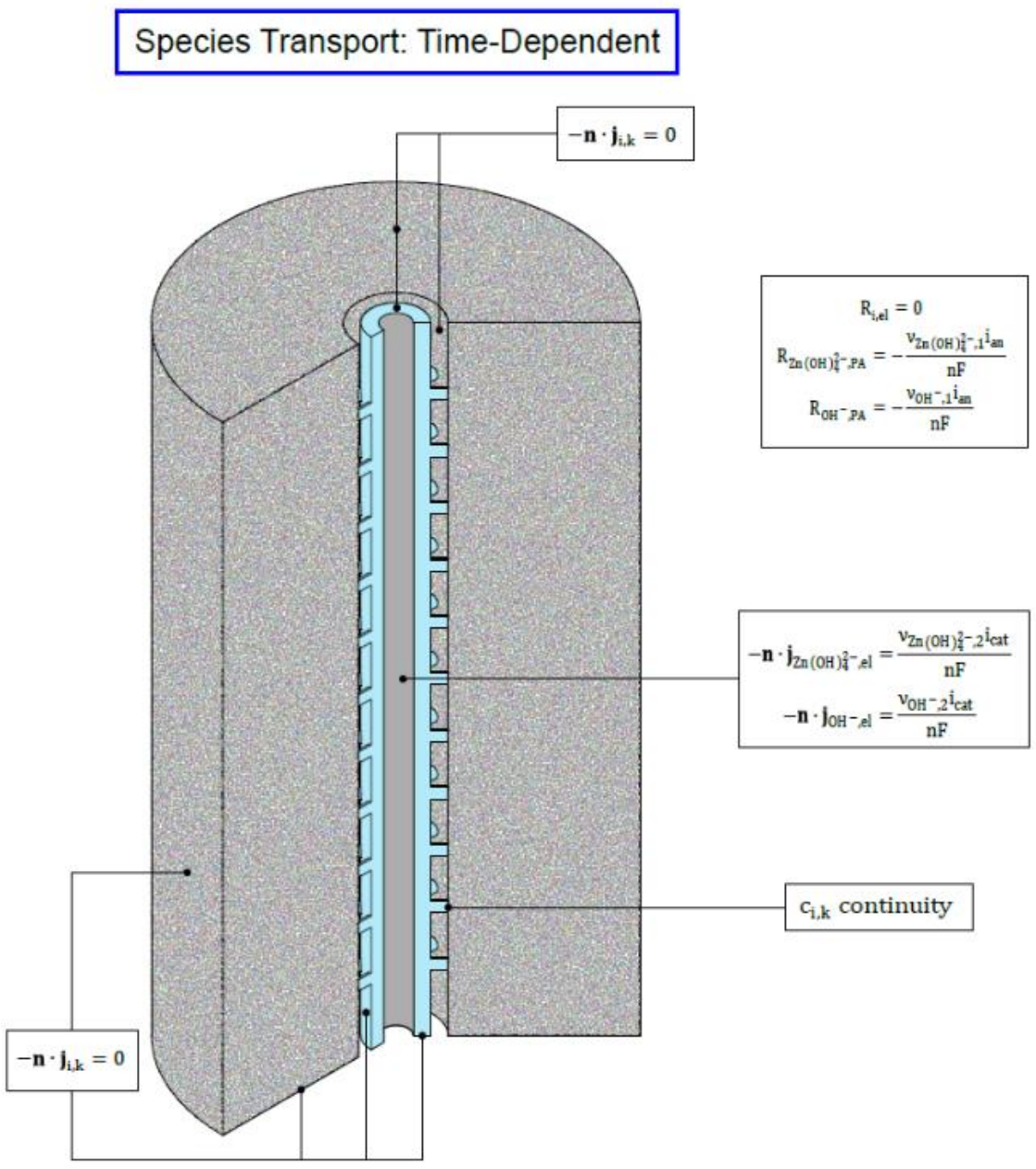

Figure 12: Electrochemical reaction terms and species conservation boundary conditions for the transient model.

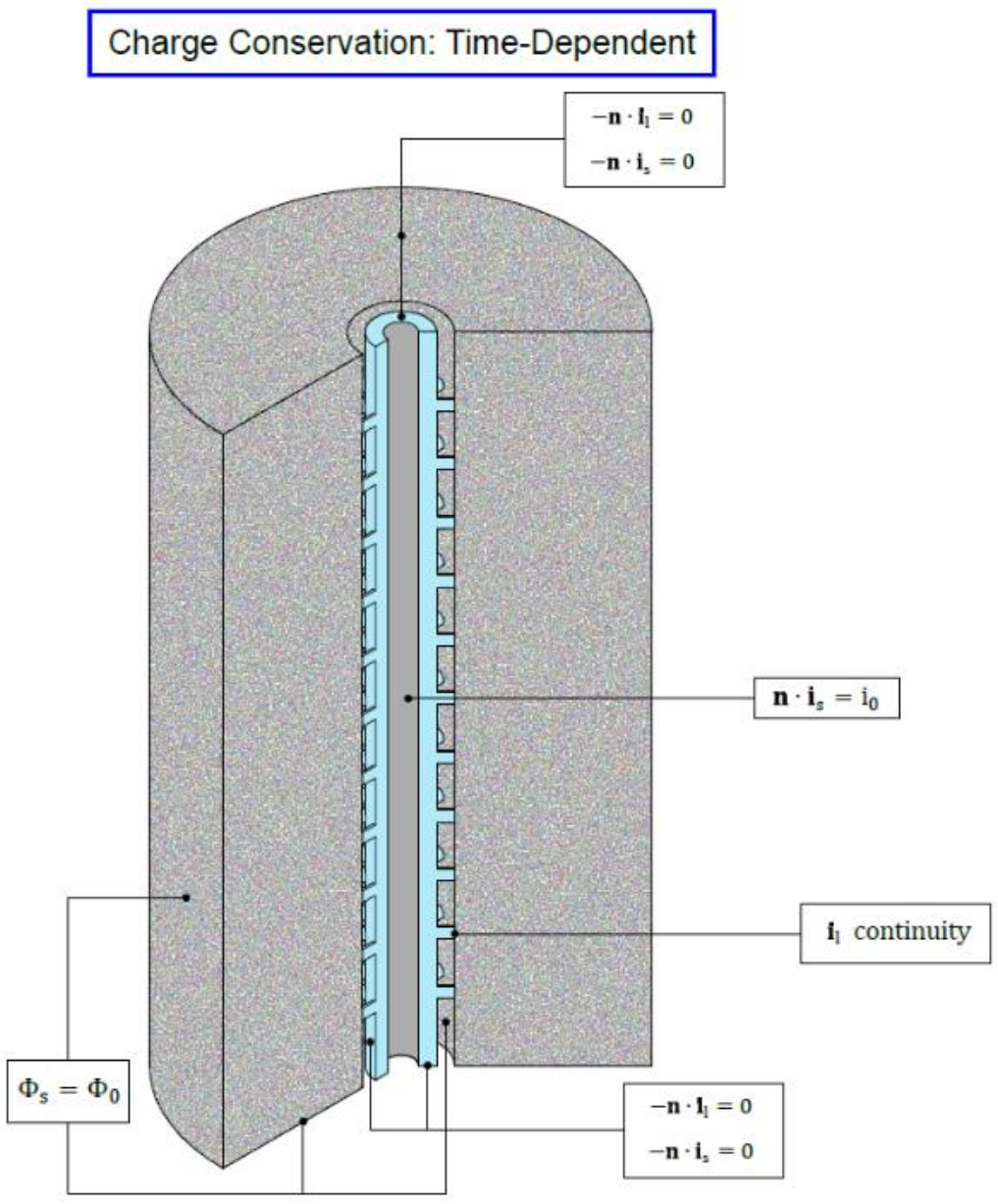

Figure 13: Charge-conservation boundary conditions for the transient model.

5.2 Electrochemical reaction terms and boundary conditions for the steady-state model

Since the only anodically active region is the external border of the porous electrolyte domain, on the one hand, we set the volumetric reactions rates in the porous medium equal to zero:

$$R_{i,el}=0$$

$$R_{i,PA}=0$$

Eq. S16

and, on the other hand, the chemical and electrical anodic boundary conditions are coupled:

$$-\mathbf{n}\cdot\mathbf{j}_{Zn(OH)_4^{2-},PA} = \frac{\nu_{Zn(OH)_4^{2-},1}\, i_{an}}{nF}$$

$$-\mathbf{n}\cdot\mathbf{j}_{OH^-,PA} = \frac{\nu_{OH^-,1}\, i_{an}}{nF}$$

Eq. S17

Moreover, the boundary conditions for the charge-conservation problem are modified as follows:

$$\begin{cases} -\mathbf{n}\cdot\mathbf{i}_l = 0, \qquad -\mathbf{n}\cdot\mathbf{i}_s = 0 \quad \text{insulation} \\ \Phi_s = \Phi_0 \quad \text{porous anode (right wall)} \\ \qquad \mathbf{n}\cdot\mathbf{i}_s = i_0 \quad \text{rod surface} \end{cases}$$

Eq. S18

Electrochemical reaction terms and electrical boundary conditions for the transient-state model are depicted in Figure 14 and Figure 15, respectively.

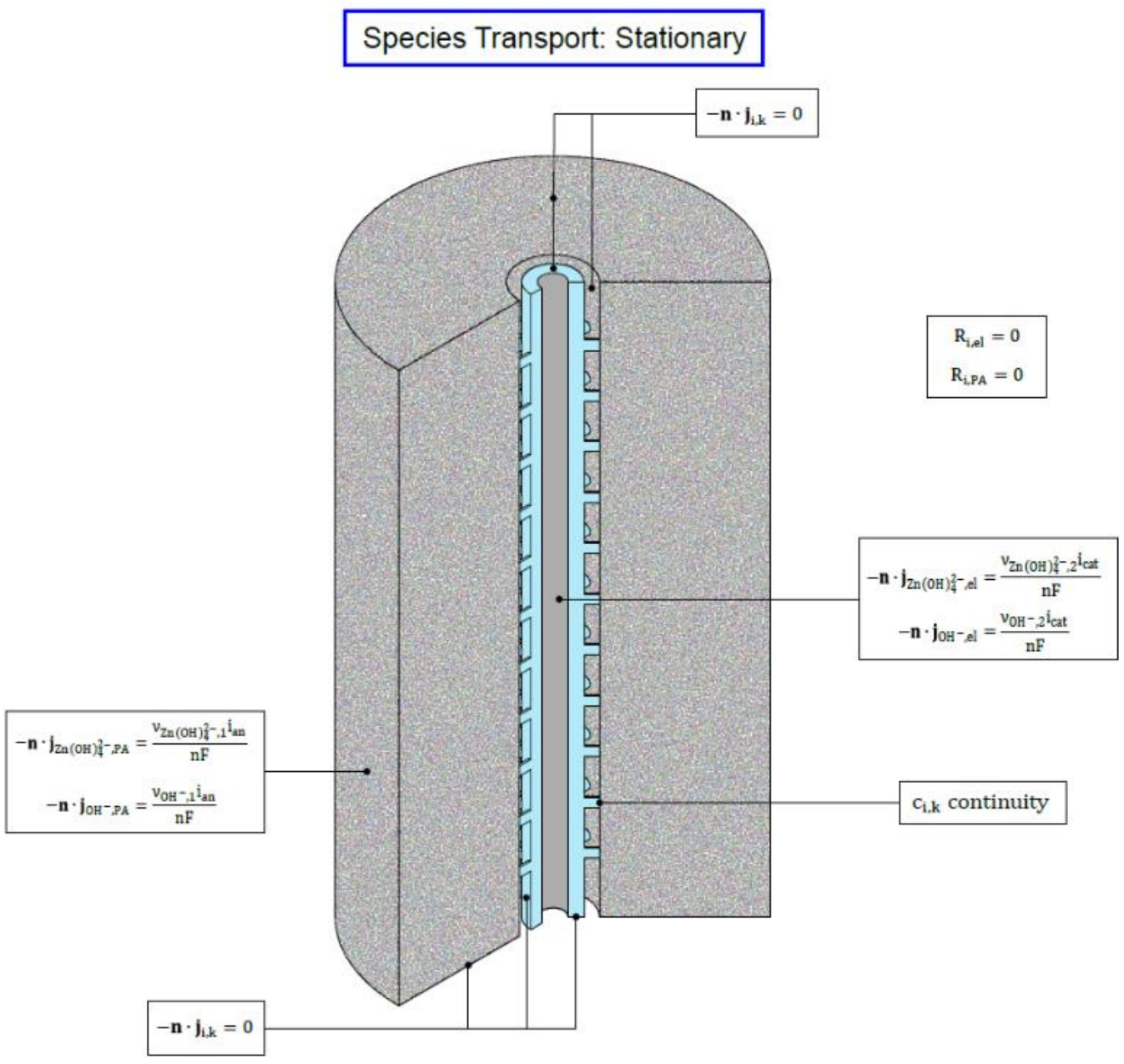

Figure 14. Electrochemical reaction terms and species conservation boundary conditions for the transient model.

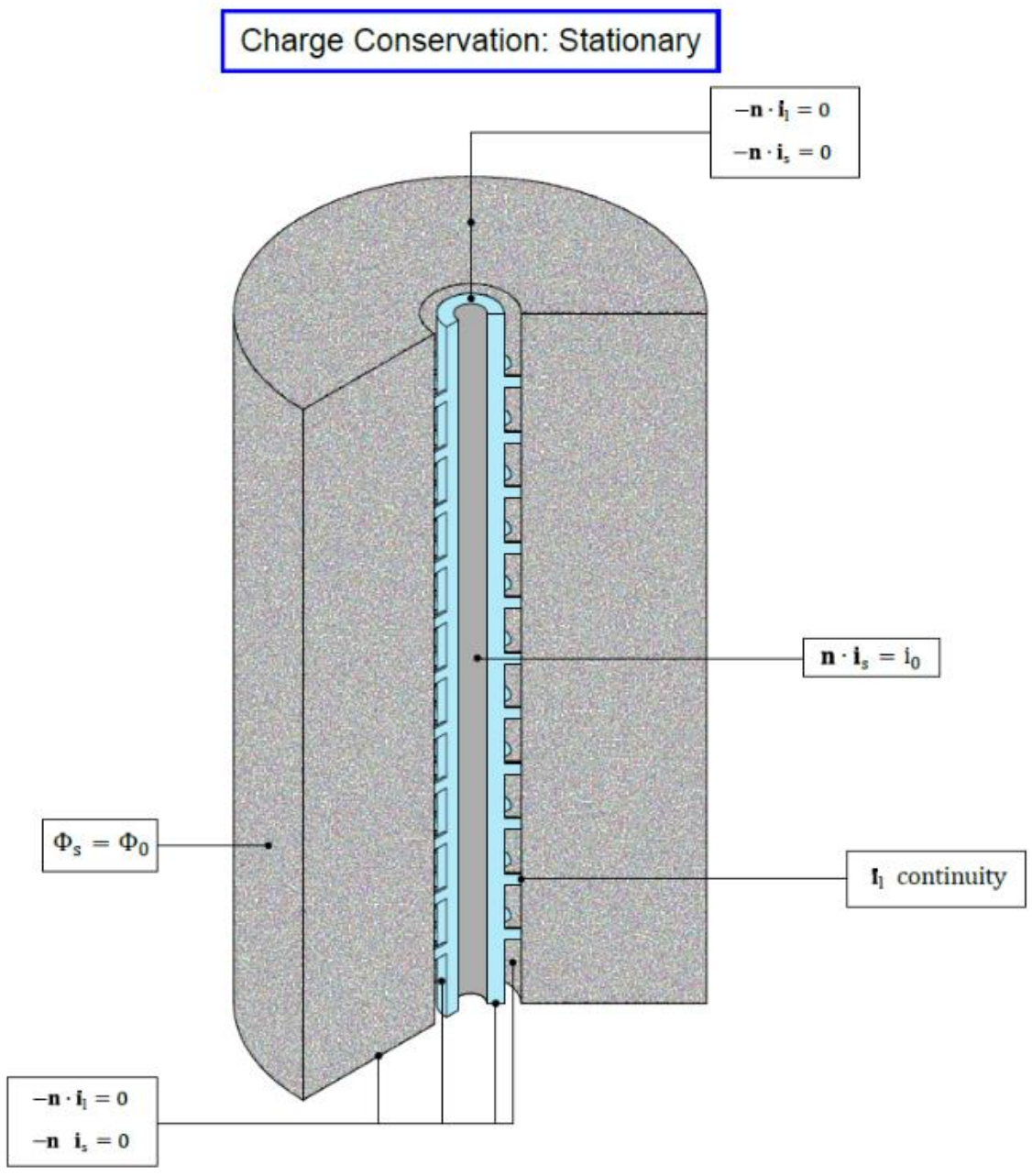

Figure 15. Charge-conservation boundary conditions for the transient model.

6. Results of simulations

The normalized computed concentration profile of zincates, obtained with the simulation described in Section 5, is reported in Figure 16. This profile shows a minimum in the porous anode domain, deriving from the accumulation of precipitated zincates within the particulate anode bed close to the border with the free-electrolyte zone, due to locally higher current densities in the initial stages of anode discharge. Precipitation of zincates leads to progressive passivation of the Zn particles in the high-current density region, that confines the electrochemical reactivity towards the outer wall, thus leading to a steady state profile in which the concentration of zincates decreases from the external wall towards the free-electrolyte region.

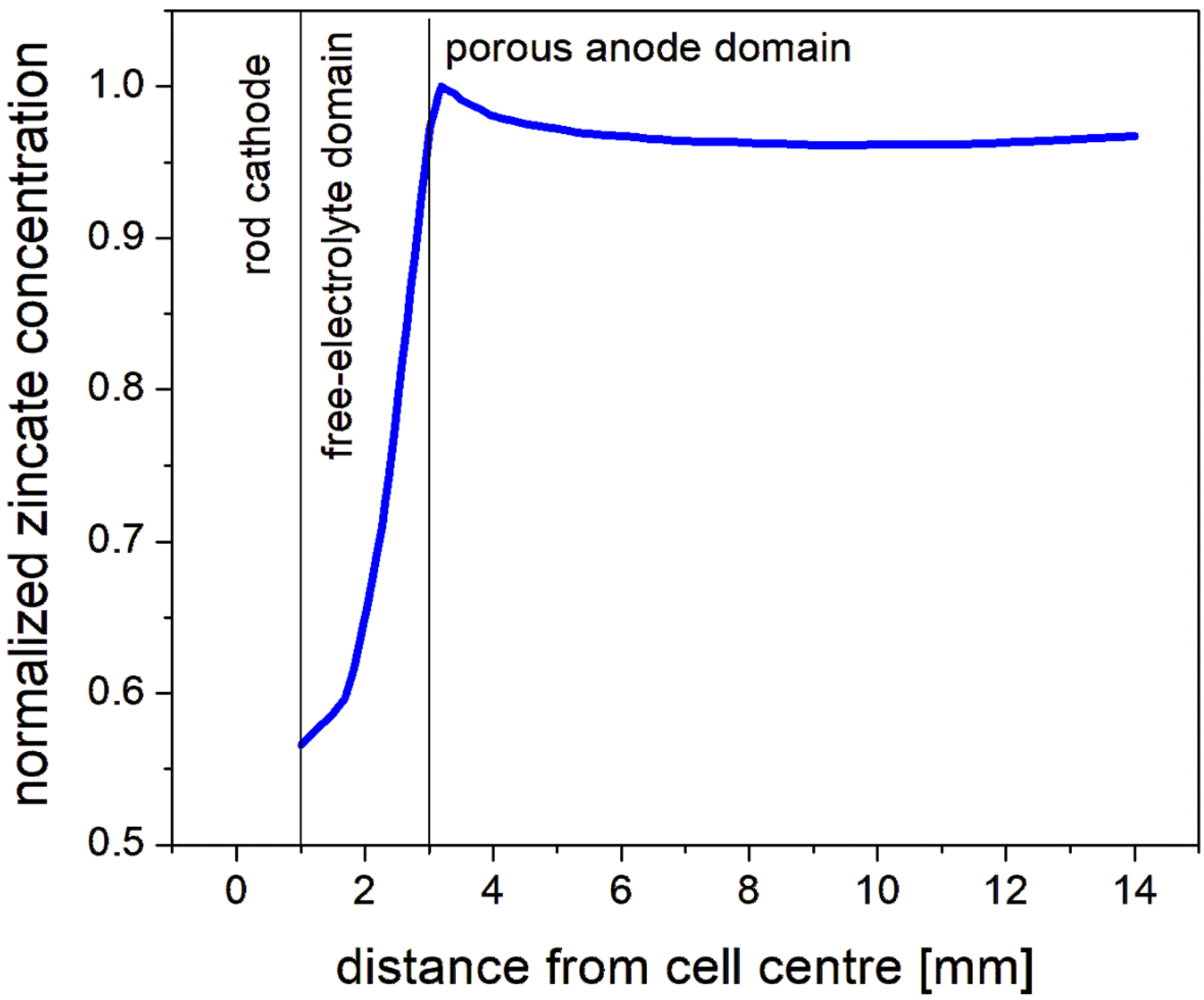

Figure 16. Calculated zincate concentration profie.

Parameter	Value	Unit	Definition	Ref.
σ_{el}	62.66	S m^{-1}	Electrical conductivity of electrolyte	[2]
σ_s	16.9 10^6	S m^{-1}	Electrical conductivity of solid phase (Zn)	
ε	0.38	-	Porosity	
$D_{OH^-,el}$	4.5 10^{-9}	m^2 s^{-1}	Diffusion coefficient of hydroxide ion	[3]
$D_{Zn(OH)_4^{2-},el}$	7.1 10^{-9}	m^2 s^{-1}	Diffusion coefficient of zincate ion	[4]
i_{time}	0.85	A	Current imposed in transient simulation	
i_{steady}	0.67	A	Current imposed in stationary simulation	
Φ_s	-1.225	V	Porous anode boundary condition	
$\nu_{Zn(OH)_4^{2-}}$	-1	-	Stoichiometric number for zincate ion	

ν_{OH^-}	4	-	Stoichiometric number for hydroxide ion	
$z_{Zn(OH)_4^{2-}}$	-2	-	Charge number of zincate ion	
z_{OH^-}	-1	-	Charge number of hydroxide ion	
n	2	-	Number of electrons involved in reaction	
$a_{a,Zn}$	0.5	-	Anode transfer coefficient of zinc electrode	5
$a_{c,Zn}$	0.5	-	Cathode transfer coefficient of zinc electrode	5
$i_{0,Zn}$	10	mA cm^{-2}	Exchange current density of zinc	5
$E^0_{eq,Zn}$	-1.25	$V_{vs. SHE}$	Standard potential of zinc corrosion at 25°C	
$\Phi_{l,0}$	$-E^0_{eq,Zn}$	V	Initial electrolyte potential	
$\Phi_{s,0}$	0	V	Initial electrode potential	
c_{0,OH^-}	6	mol L^{-1}	Initial hydroxide ion concentration in electrolyte	
$c_{0,Zn(OH)_4^{2-}}$	0.2	mol L^{-1}	Initial zincate ion concentration in electrolyte	
t	[0;100]	min	Range of time for time-dependent simulation	

Table 1 – Numerical values of constants and parameters used for simulations.

References

1. Bozzini, B. & Sgura, I. A conceptual, mathematical and quantitative reassessment of the Thin-film Flooded Agglomerate Model for air cathodes. *Journal of Electroanalytical Chemistry* 950, 117855 (2023).

2. GILLIAM, R., GRAYDON, J., KIRK, D. & THORPE, S. A review of specific conductivities of potassium hydroxide solutions for various concentrations and temperatures. *Int J Hydrogen Energy* 32, 359–364 (2007).

3. Deiss, E., Holzer, F. & Haas, O. Modeling of an electrically rechargeable alkaline Zn–air battery. *Electrochim Acta* 47, 3995–4010 (2002).

4. Jung, C.-Y., Kim, T.-H., Kim, W.-J. & Yi, S.-C. Computational analysis of the zinc utilization in the primary zinc-air batteries. *Energy* 102, 694–704 (2016).

5. Yu, W. *et al.* Elucidating the mechanism of discharge performance improvement in zinc-air flow batteries: A combination of experimental and modeling investigations. *J Energy Storage* 40, 102779 (2021).
